# Unilateral Lens Coloboma With Congenital Cataract: A Case Report From Central India

**DOI:** 10.7759/cureus.43051

**Published:** 2023-08-06

**Authors:** Rajesh Pattebahadur, Kanishk Singh, Puja Bang

**Affiliations:** 1 Ophthalmology, All India Institute of Medical Sciences, Nagpur, Nagpur, IND

**Keywords:** complicated cataract surgery, developmental cataract, mixed amblyopia, lens coloboma, paediatric cataract

## Abstract

A ten-year-old male child was referred with complaints of blurring of vision and deviation of the eye. On examination, the right eye has an esodeviation squint with a best corrected visual acuity of 6/60 Snellen's acuity and 6/6 Snellen's acuity in the left eye. Slit-lamp biomicroscope of the right eye showed coloboma at the 9 o'clock position with cataract. The rest of the anterior and posterior segments was normal in both eyes. Thus, a diagnosis of unilateral lens coloboma with amblyopia was made.

## Introduction

A lens coloboma is a condition that occurs due to abnormal zonules or the complete absence of zonules. This is seen as either a flattening of the equator or the lens equator may show a notch in the region where the zonular defect is present [1]. Unlike a true coloboma, there is no actual loss of tissue in lens coloboma; instead, the lens becomes thicker and more spherical due to the unequal pull of the zonular fibres. The most common site is the inferonasal quadrant, which corresponds to the site of the embryonic fissure [1]. Lens coloboma can be isolated in one eye or both eyes, and it can also present with uveal coloboma. Lens coloboma is also associated with cataract and can lead to visual deprivation in cases where it is significant in density or if its location is in the optical region of the lens [1].

## Case presentation

**Figure 1:**
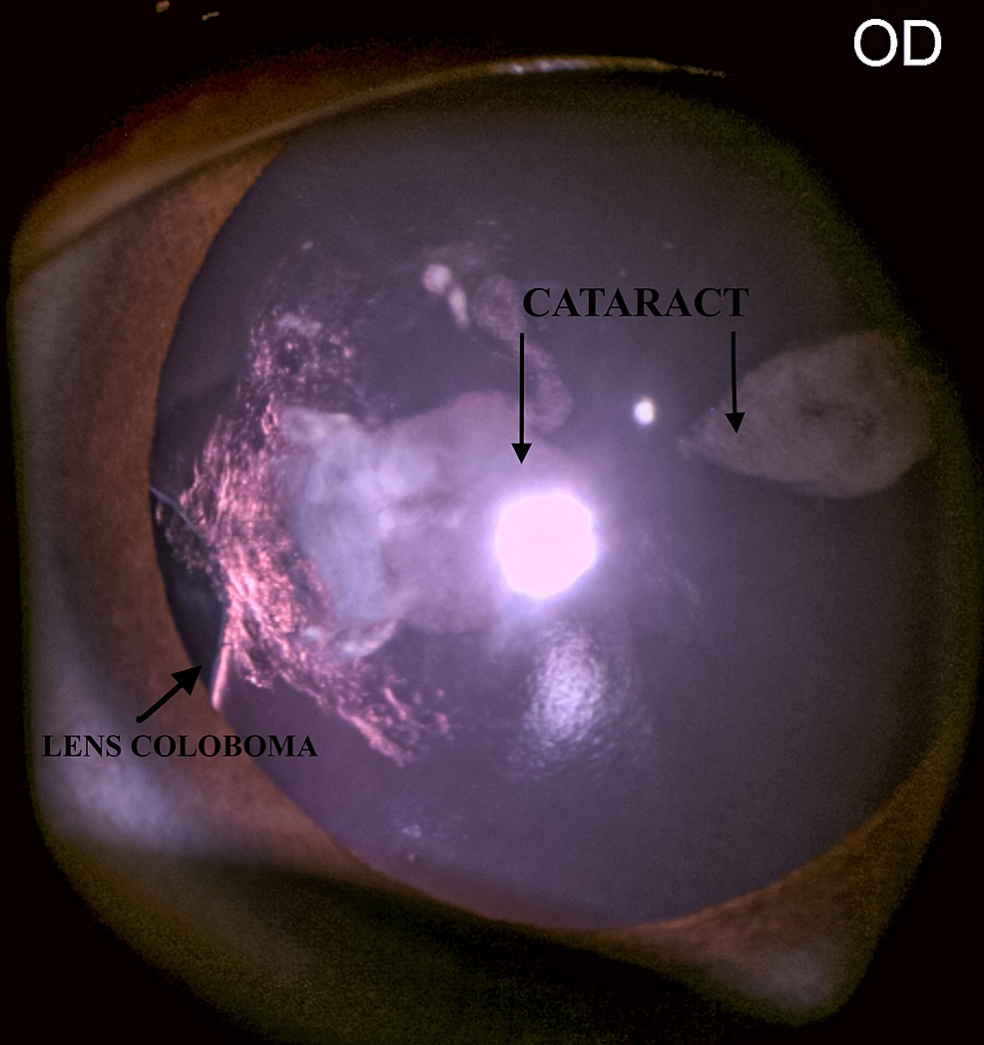
Right eye slit-lamp image Right eye slit-lamp image showing lens coloboma and congenital cataract.

A 10-year-old male child was referred with a complaint of blurring of vision and deviation of the eye. On examination, the right eye had an iso-deviation squint of approximately 30 degrees. The best-corrected vision for OD (oculus dexter or right eye) was 6/60, & for OS (oculus sinister or left eye) was 6/6. On dilatation, the right eye was showing a cataract with lens coloboma at around the 9 o'clock position. Zonular status was broken; still, the slit-lamp examination was difficult as the patient was uncooperative and had nystagmus. The location of the cataract was in the central optical area, leading to amblyopia (Figure 1).

**Figure 2:**
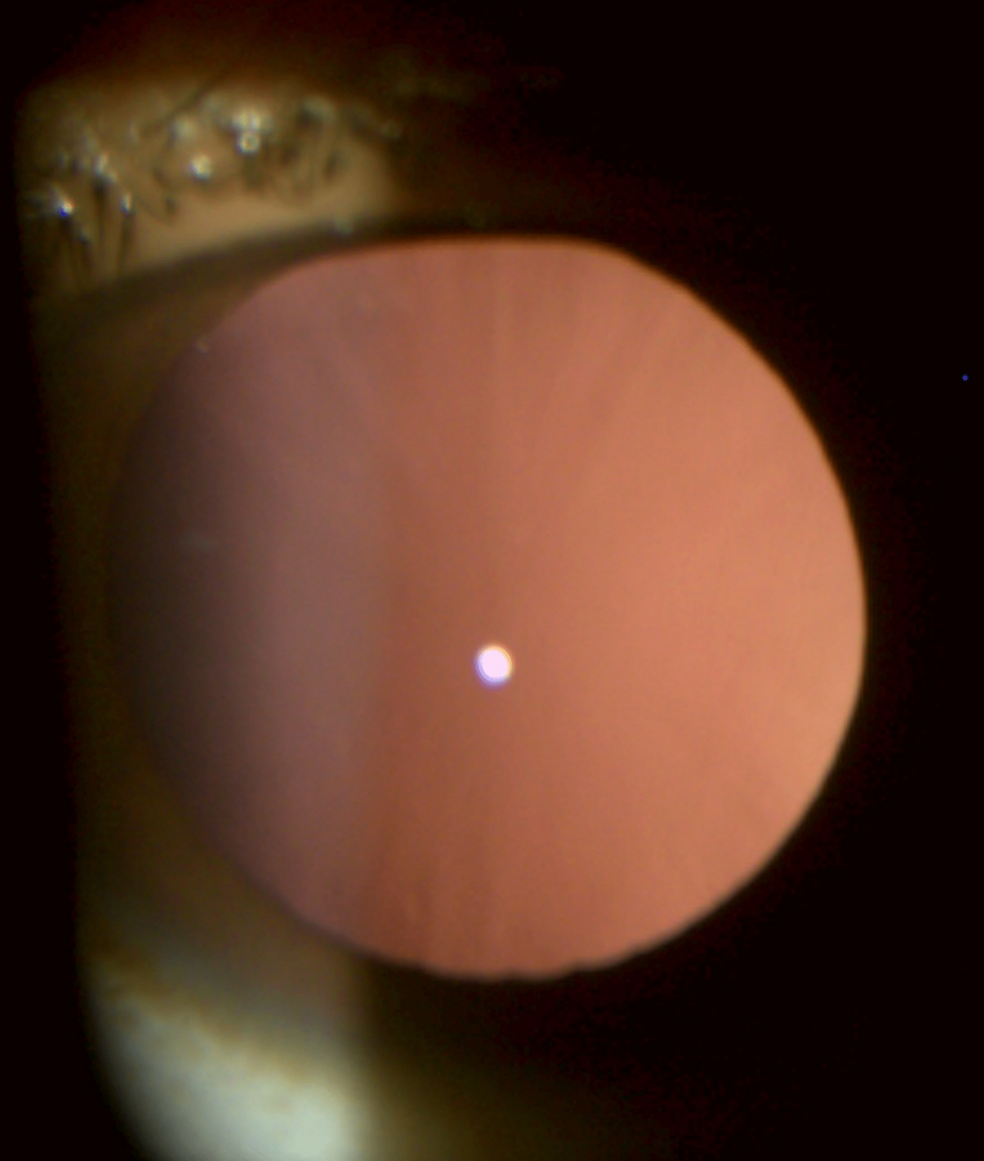
Left eye slit-lamp image Left eye slit-lamp image showing no coloboma and no cataract.

The left eye was showing an anterior segment within normal limits, without any lens opacity or coloboma (Figure 2).

In both eyes, iridodonesis or phacodonesis was absent. Fundus examination of both eyes was within physiological limits. There were no associated systemic diseases or positive family history noted in the case. Thus, the final diagnosis of OD-isolated lens coloboma with cataract and amblyopia was made. Amblyopia was the result of the sensory deprivation caused by cataract present in the pupillary area. The patient was planned for OD lens aspiration with posterior chamber intraocular lenses (PCIOL) followed by amblyopia therapy. Due to financial problems, the patient chose to opt for another hospital for further treatment, so follow-up records are not available.

## Discussion

The failure of foetal fissure closure during the development of ocular structure will lead to the formation of lens coloboma. These are seen unilaterally in the majority of cases. Lens coloboma is not an actual coloboma - there is no defect in the lens tissue. In these cases, the ciliary body or zonule does not develop, which releases the tension on the lens capsule. This further leads to the formation of the notch in an area corresponding to an absent zonule or ciliary body. Thus, it should be labelled as zonular coloboma [1].

Sometimes lens coloboma can present as defects in other ocular structures, like the optic disc, causing optic disc coloboma; the choroid, leading to choroidal coloboma; or the iris, leading to iris coloboma [2-3]. Such patients are more susceptible to the development of cataracts. Also, there are higher chances of retinal detachment in these patients, with the nasal quadrant being the most common site for retinal detachment. It has been hypothesised that a ciliary body abnormality is inducing abnormal adherence between the lens and peripheral retina in these cases, causing retinal detachment [4]. A break in the ciliary body can also give access to fluid, leading to detachment of the retina. A few systemic diseases, like Marfan syndrome and Marshall syndrome, can have an association with lens coloboma [4]. Lens coloboma has been associated with Marfan syndrome in many case reports in the literature. The elevated levels of transforming growth factor β might be responsible for this [5]. In the presenting case, there was unilateral lens coloboma in the right eye with congenital cataract leading to amblyopia. The patient did not have any other systemic diseases. 

Accurate evaluation of the anatomy of the lens, zonulae, and ciliary body is essential for the successful surgical treatment of such cases. Some in vivo studies of the human zonular apparatus and lens have been done using high-resolution ultrasound biomicroscopy. The use of Scheimpflug imaging principle-based images can depict a more accurate view of the lens coloboma morphology and can help in anticipating potential complications during surgery [6]. As our patient was a child, he was not cooperative. So a Pentacam® (Oculus Optikgeraete GmbH, Wetzlar, Germany) examination was not done.

If lens coloboma is associated with cataract, then treatment mainly involves surgical intervention. This is mainly done to avoid the risk of amblyopia. In lens coloboma patients, doing surgery is extremely challenging. The surgeon needs to be aware and avoid complications like aspiration of capsular fornix while doing lens aspiration; there could be extension dialysis of zonules; vitreous may come into the anterior chamber; and there could be intraocular lens decentration. Therefore, the use of a capsular tension ring (CTR) is advisable before phacoemulsification, so the capsular bag deformity could be prevented. Besides, CTR will also help in nucleus rotation and preventing capsular collapse, which can further complicate the surgery [7]. The treatment will also need follow-ups for treating the underlying amblyopia, if present.

## Conclusions

Lens coloboma is an uncommon condition in which a change in lens shape is caused by a defect in zonules. If present on the unilateral side, it can cause amblyopia at an early age. So, knowledge about lens coloboma and its associated abnormalities can help ophthalmologists make early diagnoses. Surgical treatment is challenging, but various approaches for its management can help in avoiding intra-operative and post-operative complications. As such patients can develop amblyopia and strabismus due to associated cataracts, amblyopia therapy and treatment of strabismus become essential parts of the treatment to achieve maximum visual recovery.
